# Supplementary material for: Hetero-pentamerization determines mobility and conductance of Glycine receptor α3 splice variants
Source: Cell Mol Life Sci. 2022 Oct 5;79(11):540. doi: 10.1007/s00018-022-04506-9 (PMC9534812; doi:10.1007/s00018-022-04506-9)
Supplement: Supplementary file 1 — Supplementary file1 (DOCX 6257 KB) [file 18_2022_4506_MOESM1_ESM.docx]

# Supplementary information to:

**Hetero-pentamerization determines mobility and conductance
of Glycine receptor α3 splice variants**

Veerle Lemmens^1,2,^*, Bart Thevelein^1,^*, Yana Vella^6^, Svenja Kankowski^3,4^, Julia Leonhard^4^, Hideaki Mizuno^5^, Susana Rocha^2^, Bert Brône^6^, Jochen C. Meier^4,#^, Jelle Hendrix^1,2,#^

^1^ Dynamic Bioimaging Lab, Advanced Optical Microscopy Centre and Biomedical Research Institute, UHasselt, B-3590 Diepenbeek, Belgium.

^2^ Molecular Imaging and Photonics, Chemistry Department, KU Leuven, B-3001 Heverlee, Belgium.

^3^ Institute of Neuroanatomy and Cell Biology, Hannover Medical School, D-30625 Hannover, Germany

^4^ Division Cell Physiology, Zoological Institute, Technical University Braunschweig, D-38106 Braunschweig, Germany.

^5^ Laboratory of Biomolecular Network Dynamics, Biochemistry, Molecular and Structural Biology Division, Department of Chemistry, KU Leuven, B-3001 Heverlee, Belgium

^6^ Neurophysiology Lab, Biomedical Research Institute, UHasselt, B-3590 Diepenbeek, Belgium.

* These authors contributed equally.

^#^ Correspondence:

| [Jochen.meier@tu-braunschweig.de](mailto:Jochen.meier@tu-braunschweig.de)  Technische Universität Braunschweig  Spielmannstraße 7  38106 Braunschweig  Tel.: +49 531 391 3254 | [Jelle.hendrix@uhasselt.be](mailto:Jelle.hendrix@uhasselt.be)  Hasselt University  Agoralaan C (BIOMED)  B-3590 Diepenbeek  Tel. +32 11 269213 |
| --- | --- |

# Supplementary figures

[Supplementary Figure S1 – Immunocytochemistry of FP tagged GlyR 3](#_Toc84020694)

[Supplementary Figure S2 – Combined fluorescence microscopy and electrophysiology 4](#_Toc84020695)

[Supplementary Figure S3 – Whole cell patch clamp electrophysiology 5](#_Toc84020696)

[Supplementary Figure S4 – Control experiments for image correlation spectroscopy 6](#_Toc84020697)

[Supplementary Figure S5 – Experimental optical resolution of the used microscopes 8](#_Toc84020698)

[Supplementary Figure S6 – Outside-out patch clamp electrophysiology. 9](#_Toc84020699)

[Supplementary figure S7 – Digital filtering of on-cell time traces 10](#_Toc84020700)

[Supplementary figure S8 – Intensity-dependence of fluctuation experiments. 11](#_Toc84020701)

# Supplementary tables

[Supplementary Table S1 – RICS and TICS dynamic ROI analyses 12](#_Toc84006134)

[Supplementary Table S2 – TICS and TICCS static ROI analyses 12](#_Toc84006135)

[Supplementary Table S3 – Pearson’s correlation coefficient 13](#_Toc84006136)

[Supplementary Table S4 – Single-step photobleaching analyses 13](#_Toc84006137)

[Supplementary Table S5 – Brightness of eGFP-tagged proteins in HEK293 cells 13](#_Toc84006138)

# Supplementary figures

## Supplementary Figure S1 – Immunocytochemistry of FP tagged GlyR


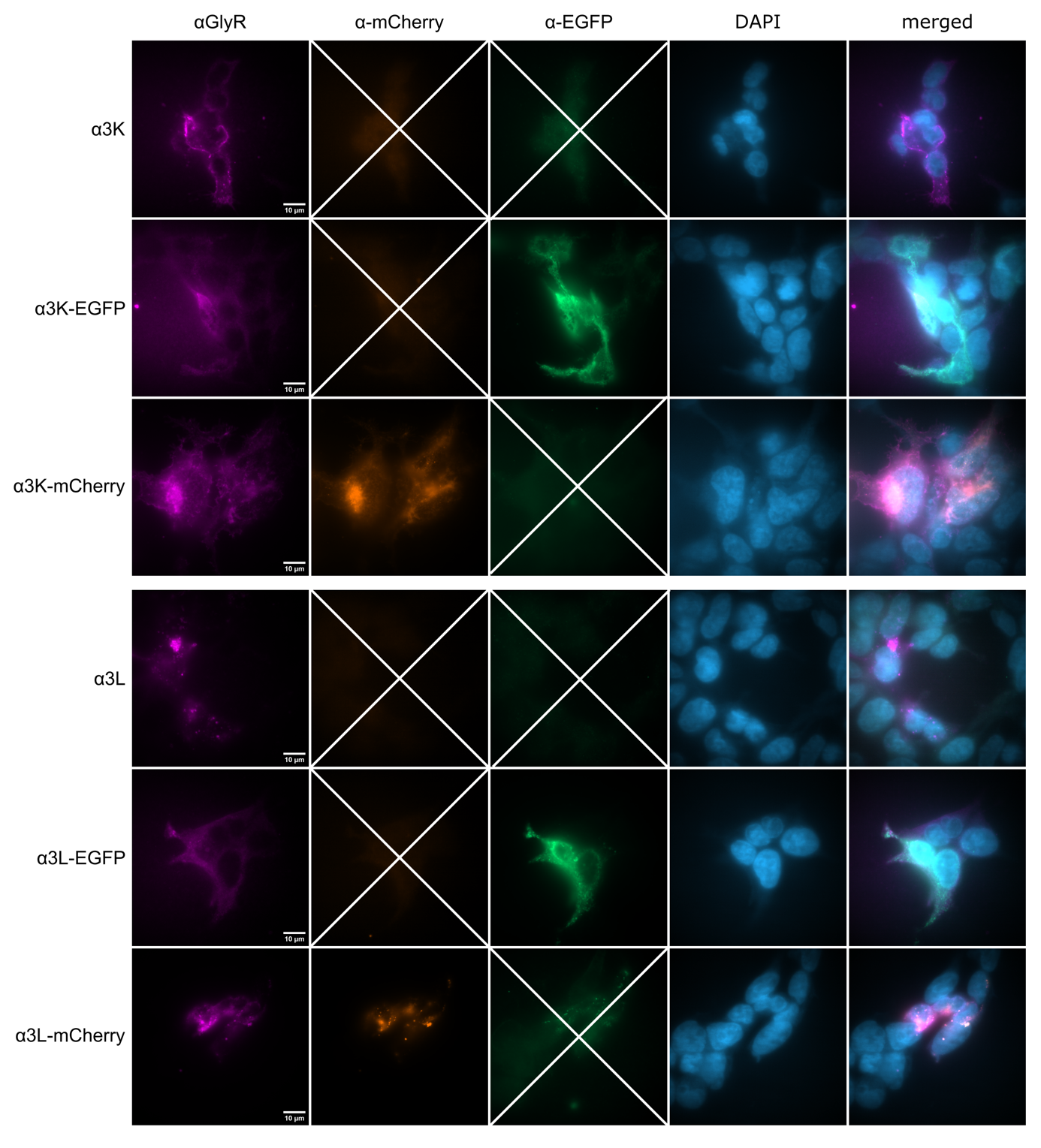


**Supplementary Fig. S1 –** Immunocytochemistry of HEK293 cells transiently expressing the different GlyR-α3 constructs as indicated on the left. Untagged α3K and α3L constructs are from references 1 and 2, respectively. The images with a white cross show the background fluorescence in the respective channels. Scale bars are 10 µm. For more information the reader is referred to the Materials and Methods section.

## Supplementary Figure S2 – Combined fluorescence microscopy and electro-physiology


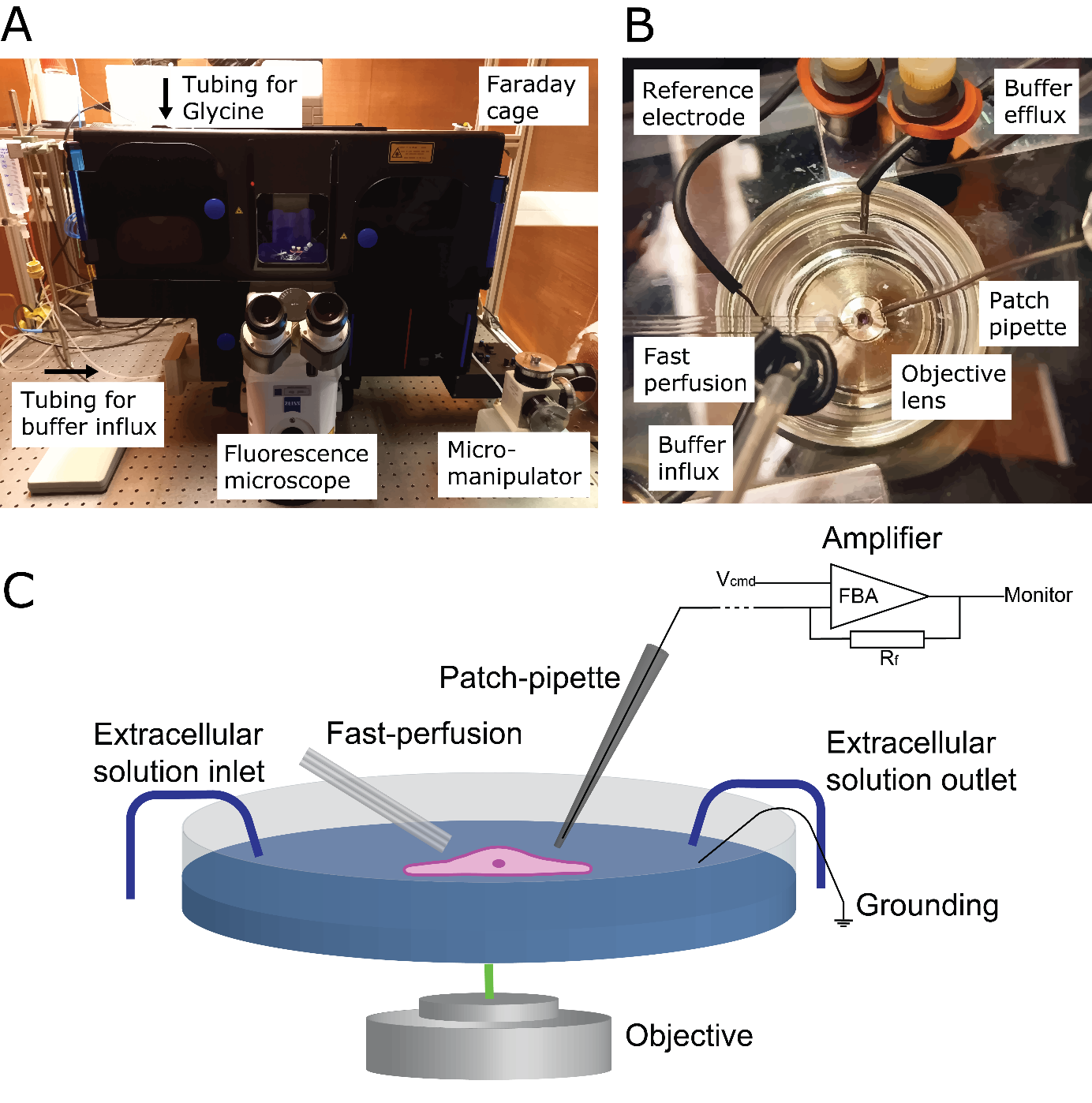


**Supplementary Fig. S2 –**. A) Overview of the setup B) Picture showing the microscope objective in the middle with the various electrophysiology components around it. C) Schematic of the electrophysiology setup, the fast-perfusion system is only used for the whole-cell configuration.

## Supplementary Figure S3 – Whole cell patch clamp electrophysiology


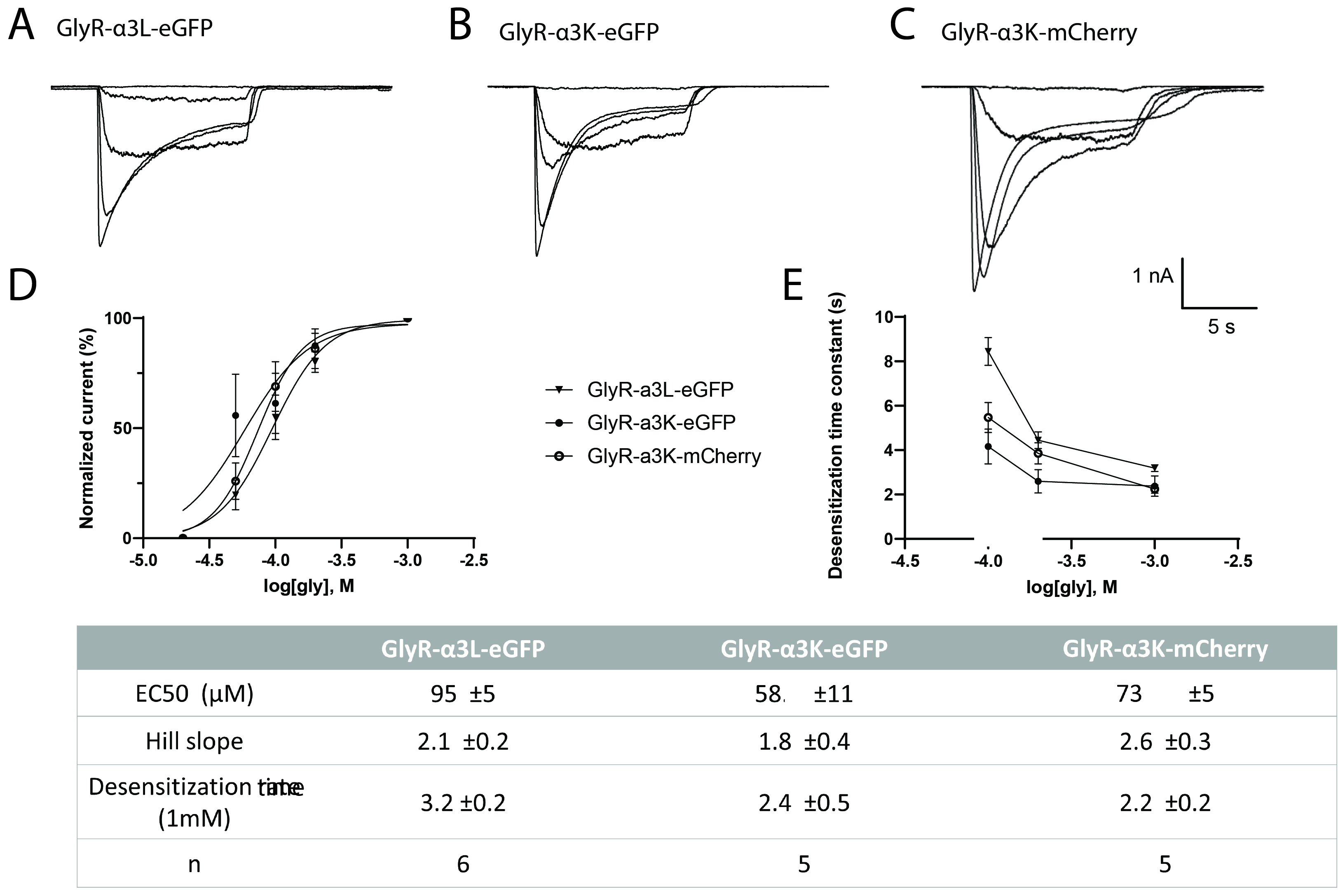


**Supplementary Fig. S3** **–** Representative traces of electrophysiological current responses of HEK293 cells transfected with plasmids encoding **(A)** GlyR-α3L-eGFP, **(B)** GlyR-α3K-eGFP and **(C)** GlyR-α3K-mCherry recorded using whole cell patch clamp. Currents were induced by 20, 50, 100, 200 and 1000 µM glycine*.* **(D)** Mean normalized current values ± SEM are plotted. Solid lines are fits to a Hill equation to obtain the half-maximal responses (EC_50_) and Hill slopes as indicated in the table below. Nikolic et al. (1998) reported average EC50 values of 54 μM and 64 μM for untagged human GlyR-α3L and GlyR-α3K, respectively. Notelaers et al. (2012) reported average EC50 values of 52 μM and 47 μM for hemagglutinin-tagged GlyR-α3L and GlyR-α3K, respectively [3-5]. Adding five (bulky) fluorescent proteins to a GlyR apparently affects the electrophysiological signature slightly. **(E)** Desensitization time constants as a function of glycine concentration for GlyR-α3L‑eGFP, GlyR-α3K‑eGFP and GlyR‑α3K‑mCherry.

## Supplementary Figure S4 – Control experiments for image correlation spectroscopy


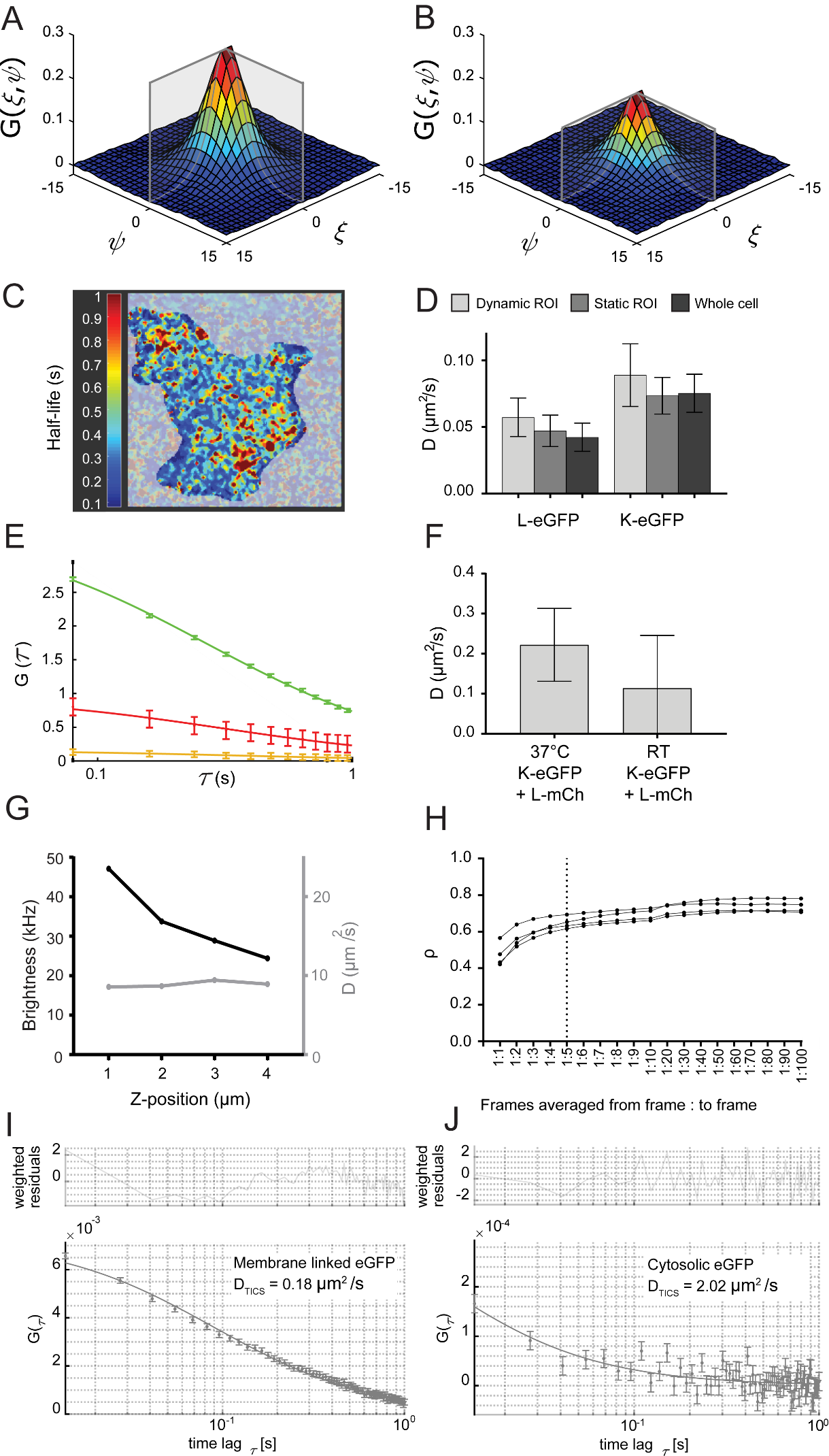


**Supplementary Fig. S4** **– A)** 2D correlation function of ROI1, supplementary to Fig. 1F. **B)** 2D correlation function of ROI1 minus ROI2, supplementary to Fig. 1F. **C)** Example of a spatially resolved TICS experiment. Shown is an image of the half-life of the TICS correlation function in each pixel, displaying regions with GlyR cluster exhibiting slow diffusion. **D)** Diffusion coefficient of GlyR-α3L eGFP and GlyR-α3K-eGFP obtained via TICS on the whole cell region (black), on the static ROI after excluding clusters with average intensity thresholding (dark grey) and on the dynamic ROI after excluding clusters with a mask dependent on the intensity in each single frame (light grey). **E)** Exemplary temporal mean autocorrelation (green and red) and cross-correlation (yellow) of a cell expressing GlyR-α3L-eGFP and Lyn-mCherry. Error bars are the 95% confidence intervals. Supplementary to Fig. 3. **F)** Temperature dependence of α3K-eGFP α3L-mCherry RICS autocorrelation data. **G)** Height dependence of RICS data of eGFP_5_, where 1 µm is axial position of maximum molecular brightness. **H)** The effect of averaging a variable number of frames for calculating the Pearson’s correlation coefficient *ρ* for 4 representative cells. When a single frame was used a low signal-to-noise in the image influences the *ρ*. When frames are averaged, the single-to-noise is increased resulting in a more constant *ρ*. We averaged 5 frames for calculation of the *ρ.* **I-J)** Diffusion measurements of (I) membrane linked eGFP (pDisplay, Thermo Fisher) and (J) cytosolic eGFP measured at 13-ms frame time.

## Supplementary Figure S5 – Experimental optical resolution of the used microscopes

**
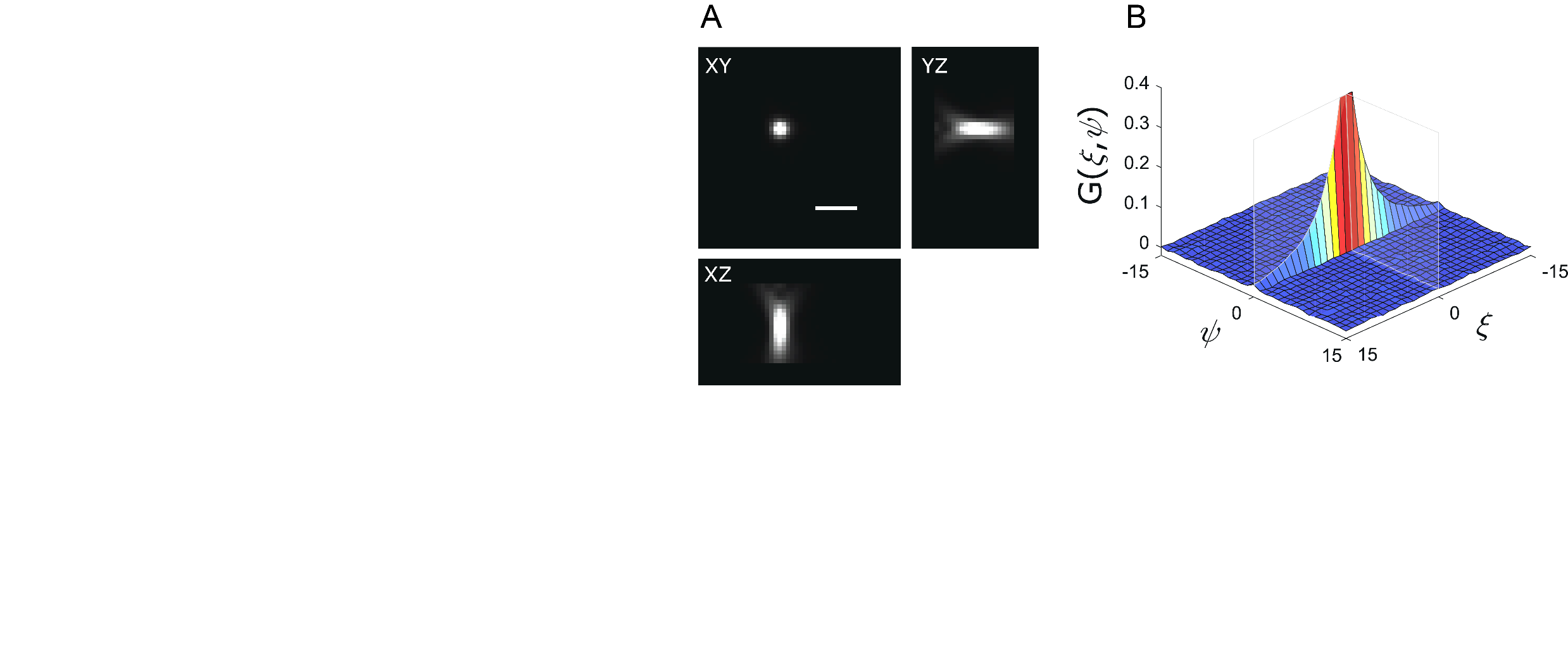
**

**Supplementary Fig. S5** – **A)** Experimental 3D point-spread function of the Elyra PS.1 microscope measured using 488-nm excitation on 20-nm fluorescent beads, used to calculate the lateral optical waist $\omega_{r}=250 nm$ for TICS experiments. Scale bar, 1 µm. **B)** RICS autocorrelation function of ATTO488-COOH, used to determine the $\omega_{r,488nm}=210 nm$ for RICS experiments (*D*_ATTO488-COOH,23°C_ = 373 µm^2^/s). For dual-color RICS experiments $\omega_{r,594nm}=220 nm$ and $\omega_{r,CCF}=215 nm$.

## Supplementary Figure S6 – Outside-out patch clamp electrophysiology.


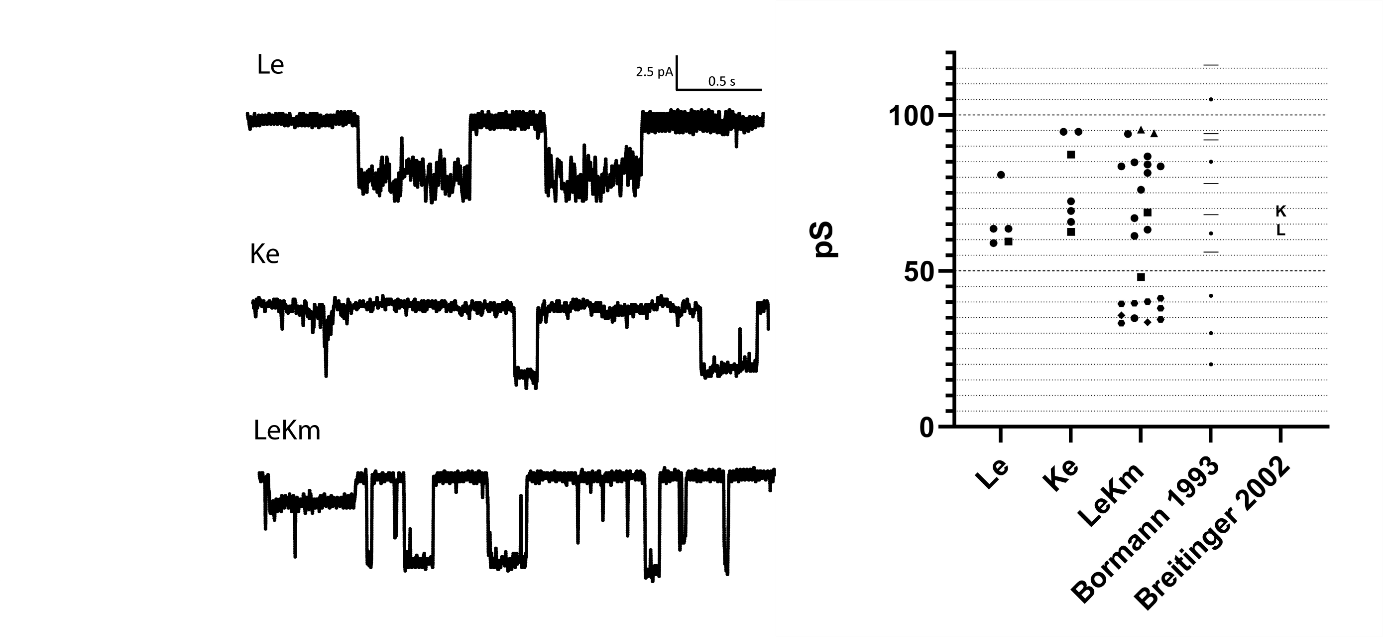


**Supplementary Fig. S6** – Left: example traces of outside-out patch clamp data filtered with a digital filter at 100 Hz. Traces were analyzed in the same way as described in figure 5B. Right: Comparison of outside-out conductance of cells transfected with the various constructs to data from literature. The data of Bormann et al. (1993) is shown as the conductance ± SD, unless n = 1. Bormann et al. did not specify whether the used construct contained ɑ3K or ɑ3L. Breitinger et al. (2002) only reported the ‘main-state conductance’, ignoring other conductance states. The letters ‘K’ and ‘L’ indicate the conductance ± SD for ɑ3K and ɑ3L respectively. Cells were transfected with either GlyR-α3L-eGFP (Le), GlyR-α3K-eGFP (Ke), or co-transfected with GlyR-α3L-eGFP and GlyR-α3K-mCh (LeKm). Recordings were performed in voltage clamp mode at RT using the same HEKA EPC10 amplifier, again using a 2.9 kHz low-pass hardware filter but with a holding potential of -60 mV. The external solution was identical to the one used in the cell-attached configuration. Patch pipettes (6-8 MΩ) were filled with intracellular solution containing 107.1 mM KCl, 10 mM Glucose, 1 mM CaCl_2_, 1 mM MgCl_2_.6Aq, 10 mM HEPES, 11 mM EGTA, 2 mM MgATP and 20 mM TEA-CL. Glycine was supplied to the outside-out patch using a fast perfusion device containing external solution with 30 µM of glycine. Analysis of outside-out recordings was done in the same way as described for cell-attached recordings.

## Supplementary figure S7 – Digital filtering of on-cell time traces


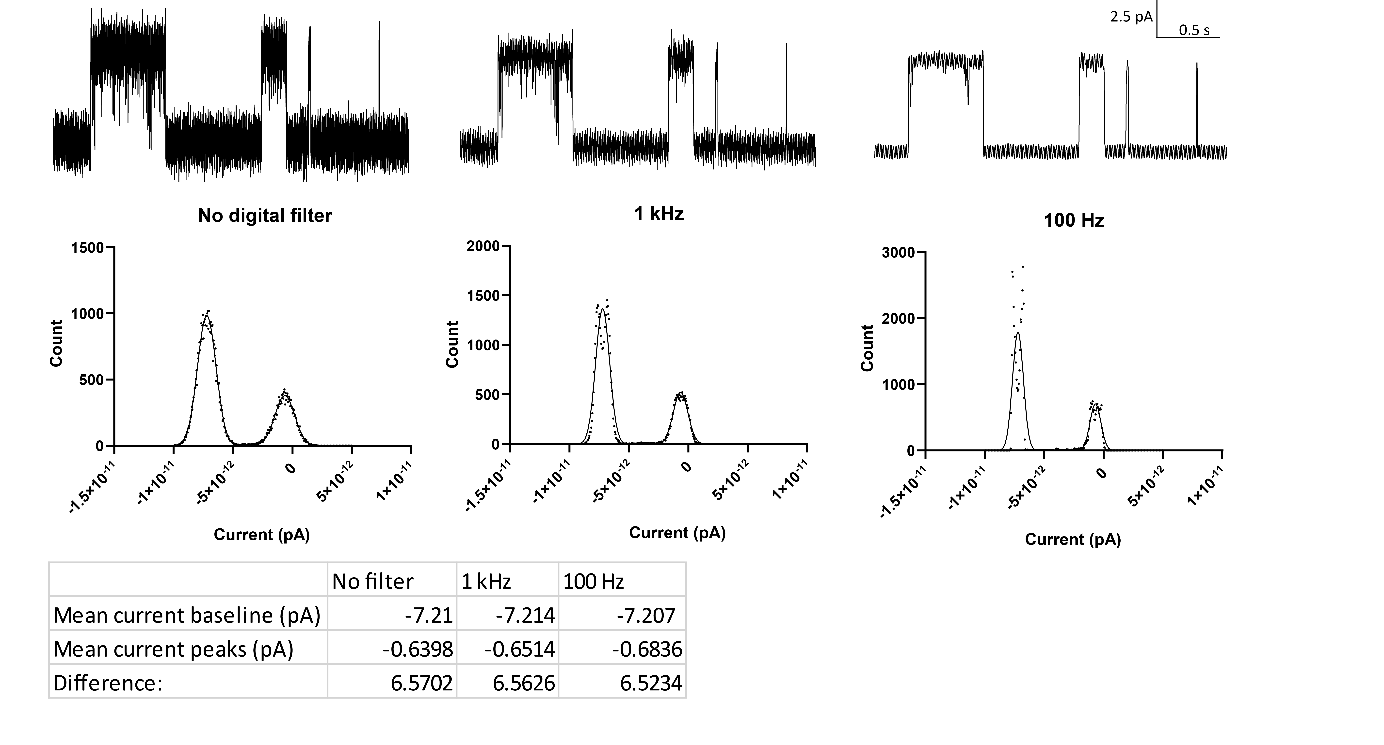


**Supplementary Fig. S7 –** Representative time trace measured in the cell-attached configuration (left) without digital filter, (middle) filtered at 1 kHz and (right) filtered at 100 Hz. Although the traces look different, the resulting current baseline and current peaks are highly similar.

## Supplementary figure S8 – Intensity-dependence of fluctuation experiments


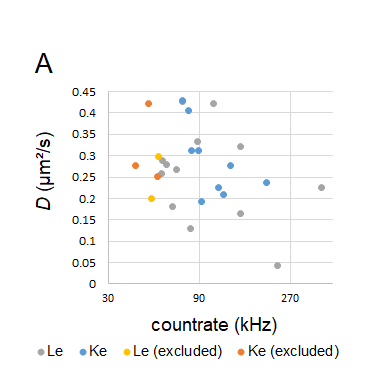

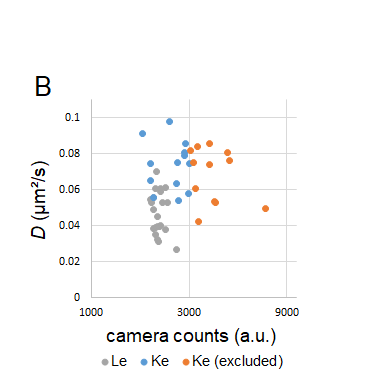


**Supplementary Fig. S8 –** Intensity-dependence of fluctuation experiments. A) Single-color RICS data of L-eGFP and K-eGFP as a function of the countrate of the timeseries. B) Single-color TICS data of L-eGFP and K-eGFP as a function of camera counts. ‘Excluded’ refers to either low signal-to-noise data (RICS) or too-high concentration data (TICS) that was omitted from the analyses in the manuscript.

# Supplementary tables

## Supplementary Table S1 – RICS and TICS dynamic ROI analyses

| **Method** | **Protein (eGFP)** | **n** | ***D* ± SD (µm^2^/s)** |
| --- | --- | --- | --- |
| **RICS** | GlyR-α3L-eGFP | 13 | 0.26 ± 0.11 |
| **RICS** | GlyR-α3K-eGFP | 9 | 0.29 ± 0.08 |
| **TICS** | GlyR-α3L-eGFP | 22 | 0.057 ± 0.014 |
| **TICS** | GlyR-α3K-eGFP | 13 | 0.089 ± 0.023 |

RICS and TICS of HEK293 cells expressing GlyR α3 eGFP isoforms with frame-based thresholding (dynamic ROI). The diffusion coefficient (*D*) is calculated as described in Materials and methods. SD = standard deviation. The number of measured cells, that were not all measured on the same day, even within experimental groups, is indicated with *n*.

## Supplementary Table S2 – TICS and TICCS static ROI analyses

| **Protein (eGFP)** | **Protein (mCherry)** | **n** | ***D* ± SD (µm^2^/s)** | |
| --- | --- | --- | --- | --- |
|  |  |  | **TICS (ACF1)** | **TICCS (CCF)** |
| GlyR-α3L-eGFP | - | 22 | 0.047 ± 0.012 | - |
| GlyR-α3K-eGFP | - | 13 | 0.074 ± 0.014 | - |
| GlyR-α3**L**-eGFP | GlyR-α3**L**-mCherry | 19 | 0.044 ± 0.011 | 0.039 ± 0.018 |
| GlyR-α3**L**-eGFP | GlyR-α3**K**-mCherry | 22 | 0.061 ± 0.011 | 0.078 ± 0.018 |
| GlyR-α3**K**-eGFP | GlyR-α3**K**-mCherry | 5 | 0.068 ± 0.013 | 0.079 ± 0.019 |

TICS and TICCS of HEK293 cells expressing GlyR α3 isoforms with average intensity-based thresholding for clusters (static ROI). The diffusion coefficient (*D*) is calculated as described in Materials and methods. The number of measured cells, that were not all measured on the same day, even within experimental groups, is indicated with *n*.

## Supplementary Table S3 – Pearson’s correlation coefficient

| **Protein (eGFP)** | **Protein (mCherry)** | **n** | ***ρ*** | |
| --- | --- | --- | --- | --- |
|  |  |  | **No clusters** | **Whole ROI** |
| GlyR-α3L-eGFP | GlyR-α3K-mCherry | 22 | 0.61 ± 0.14 | 0.59 ± 0.13 |
| GlyR-α3L-eGFP | GlyR-α3L-mCherry | 19 | 0.63 ± 0.12 | 0.68 ± 0.16 |
| GlyR-α3L-eGFP | Lyn-mCherry | 11 | 0.35 ± 0.12 | 0.27 ± 0.09 |

The *ρ* is calculated as described in Materials and methods. The number of measured cells, that were not all measured on the same day, even within experimental groups, is indicated with *n*.

## Supplementary Table S4 – Single-step photobleaching analyses

| **Heteromeric stoichiometry** | **Heteromeric  fraction (%)** | **Homomeric  fraction (%)** | **P-value** |
| --- | --- | --- | --- |
| 1:4 | 0.13 | 0.87 | 0.024 |
| 2:3 | 0.23 | 0.77 | 0.332 |
| 3:2 | 0.36 | 0.64 | 0.725 |
| 4:1 | 0.67 | 0.33 | 0.672 |

Best fitted binomial distribution function consisting of a variable heteromeric fraction with nth order and a homomeric fraction of 5th order. P-values determined with the Chi^2^-test.

## Supplementary Table S5 – Brightness of eGFP-tagged proteins in HEK293 cells

| **Protein (eGFP)** | **Protein (mCherry)** | **n** | $\varepsilon$ **± SD (kHz)** |
| --- | --- | --- | --- |
| GlyR-α3L-eGFP | - | 13 | 44 ± 19 |
| GlyR-α3L-eGFP | GlyR-α3K-mCherry | 20 | 29 ± 11 |
| Lyn-eGFP | - | 11 | 17 ± 3 |
| Lyn-eGFP | GlyR-α3K-mCherry | 5 | 18 ± 3 |

The brightness $\varepsilon$ is calculated as described in Materials and methods. The number of measured cells, that were not all measured on the same day, even within experimental groups, is indicated with *n*.

# References

1. Meier JC, Henneberger C, Melnick I, Racca C, Harvey RJ, Heinemann U, et al. RNA editing produces glycine receptor α3P185L, resulting in high agonist potency. Nat Neurosci [Internet]. 2005;8(6):736–44.
2. Eichler SA, Förstera B, Smolinsky B, Jüttner R, Lehmann T-N, Fähling M, et al. Splice-specific roles of glycine receptor α3 in the hippocampus. Eur J Neurosci [Internet]. 2009;30(6):1077–91.
3. Notelaers K, Smisdom N, Rocha S, Janssen D, Meier JC, Rigo J-M, et al. Ensemble and single particle fluorimetric techniques in concerted action to study the diffusion and aggregation of the glycine receptor α3 isoforms in the cell plasma membrane. biochimica et Biophysica Acta. 2012;1818:3131-40.
4. Breitinger, U. and H.-G. Breitinger, Augmentation of glycine receptor alpha3 currents suggests a mechanism for glucose-mediated analgesia. Neuroscience Letters, 2016. **612**: p. 110-115.
5. Nikolic Z, Laube B, Weber RG, Lichter P, Kioschis P, Poustka A, et al. The human glycine receptor subunit α3. Journal of Biological Chemistry. 1998;273(31):19708-14.
