# Supplementary material for: Hetero-pentamerization determines mobility and conductance of Glycine receptor α3 splice variants
Source: Cell Mol Life Sci. 2022 Oct 5;79(11):540. doi: 10.1007/s00018-022-04506-9 (PMC9534812; doi:10.1007/s00018-022-04506-9)
Supplement: Supplementary file 2 — Supplementary file2 (PDF 93 KB) [file 18_2022_4506_MOESM2_ESM.pdf]

We thank the editor and reviewers for assessing our work again and the time invested. Reviewer 2 asks to put into perspective the model choice for TICS data analysis. For the convenience of the Editor and Reviewer 2, we first paste the original Query by Reviewer 2 on this matter (April 30, 2022):

- "Eq. 11: At a pixel size of 160 nm, the pixel is on the order of the size of the PSF. In addition, the intensity distribution in the lateral axis is uniform in TIRFM on these scales. In this case Eq. 11 should not be the correct fit function for the temporal correlation. Would a more appropriate fit function provide better diffusion coefficient data?"

and our original Answer (June 10, 2022):

- We thank the reviewer for the oversight and have now added the lateral optical waist $r_{1/e\text{^}2}$ of the TIRF microscope in the legend of Figure S5, corresponding to a Rayleigh lateral resolution $r_{Rayleigh}$ = 2 $r(FCS,\ i.\ e.\ at$ 1/e^2) of about 350 nm. As a pixel size of 150 nm still samples such resolutions at the Nyquist criterion, we therefore think the fit function is within experimental error correct. In any case (1) throughout the paper we refrain from converting diffusion constants to absolute molecular parameters, and merely interpret statistically significant differences between conditions and (2) the data are within experimental errors described well by the employed fit functions (Fig. 2H, 3F, S4E, S4I, S4J).

New Query by Reviewer 2 regarding this matter (June 27, 2022):

- " The authors have answered most of my question. Curiously, they insist on using Eq. 11 for their TICS data fitting, despite correct models having been published several times, and state "we refrain from converting diffusion constants to absolute molecular parameters". But that is not what they say in the article (see, for instance, lines 362-363) and they consistently report "diffusion coefficients" or "molecular diffusion coefficients" for TICS. If the authors do not claim any absolute diffusion coefficients and only report relative changes, then this must be explained and made explicit. They ought to clearly indicate at the definition of Eq. 11, that the function is an approximation and should reference the correct fit models. Alternatively, they can show that the current model approximates the proper model within the margins of error or show they are proportional and explain that in the article. Or they can just use the correct model. While I understand that this is a pedantic point, I believe that in an article readers need to be clear about data and analysis."

Our Answer:

- First of all, in our previous answer we meant to say that we do not interpret diffusion constants as physical hydrodynamic parameters such as absolute molecular size, which in cells is anyway not preferred. To avoid misinterpretation in the paper, we have added the word 'apparent' in front of 'diffusion constant', where we define the constant for RICS (line 819) and TICS (line 883).
- Secondly, for fitting our TICS data, we employed Eq. 3 of the original imaging-TIRF-FCS paper applying the method to in-membrane diffusion (Kannan et al., doi.org/10.1021/ac0624546). Here, $\tau_{diff} = \omega_{xy}^2/4D$, where ω_{xy} is the $1/e^2$ lateral waist of

the PSF. In our case this value was equal to 250 nm, as determined by 3D bead imaging (Figure S5, with FWHM = 300 nm and ω_{xy} = FWHM/(sqrt(2*ln(2)). We now cite Kannan et al. properly.

- Thirdly, we do realize later literature provided more accurate models. The Reviewer is correct in saying “the intensity distribution in the lateral axis is uniform in TIRFM on these scales”, rendering, e.g., Eq. 4 from Guo et al. (doi.org/10.1002/cphc.200700611) more suitable. However, as this is effectively the same mathematical function than Eq. 3 from Kannan et al., it merely results in a scaling factor. A pixel side length of 150 nm (our experimental case) would result in values for *D* that are exactly 2.15-fold larger when analysed via Guo et al., relative to the way we performed it (Kannan et al.):

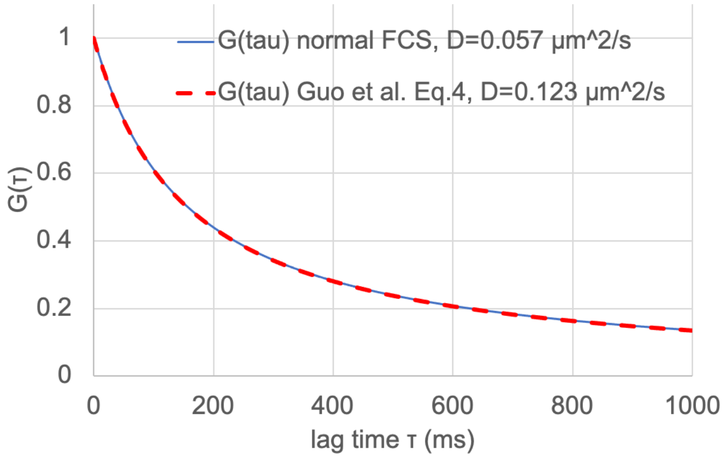

The apparent time scales of D for the glycine receptor between RICS and TICS, that originally were not orders-of-magnitude different, now thus become even slightly less so. However, one cannot compare the absolute values of D between the methods, which would require carrying out an in vitro comparative RICS and TIRF-TICS control experiment (e.g. a lipid in a supported lipid bilayer) to optimize acquisitioning and analysis conditions under which both methods render identical values for D. Although useful, we hope the reviewer can agree this is beyond the scope of this biological investigation, where mere relative differences between biological conditions are interpreted. As per the reviewer’s suggestion, we have indicated that Eq. 11 is an approximation and have referenced Guo et al.. We thank the Reviewer sincerely for insisting on this matter.
